# Exploring the Use of Mobile Health Applications in Palestinian Community Pharmacy Practice

**DOI:** 10.1016/j.curtheres.2025.100782

**Published:** 2025-03-01

**Authors:** Ahmed Nouri

**Affiliations:** Faculty of Medicine, Heinrich Heine Universität Düsseldorf, Düsseldorf, Germany

**Keywords:** Pharmacy Practice, Smartphone, Trust, Patients, Palestine

## Abstract

**Background:**

Mobile health applications have become essential tools in modern healthcare, enabling professionals to access real-time drug information, clinical guidelines, and patient management resources. While globally embraced, the adoption of these apps in resource-limited settings like Palestine remains under-researched, particularly among community pharmacists, who are pivotal to the healthcare system.

**Aims:**

This study explores the perceptions, awareness, and challenges faced by Palestinian community pharmacists regarding mobile health applications. It aims to assess the feasibility of integrating these tools into their practice to improve pharmaceutical care and patient outcomes.

**Methods:**

A cross-sectional online survey was conducted in 2023 among community pharmacists in Palestine. A self-administered electronic questionnaire was distributed via social media, targeting registered pharmacists. Data were collected using a structured, validated questionnaire addressing demographics, app usage patterns, perceived benefits, and barriers. Descriptive and inferential analyses were performed using SPSS® software, with P-values ≤0.05 considered statistically significant.

**Results:**

The study included 400 community pharmacists, predominantly female (65.8%). Pharmacists frequently used information resources for verifying drug interactions (89%) and dosages (98%), citing quick access to reliable information as a major advantage. Barriers included time constraints (92.3%) and concerns about patient trust (77.8%). No significant associations were found between demographics (e.g., gender, years of experience) and perceptions of app usefulness or trust. A strong positive correlation (*P* < 0.001) was observed between community pharmacists’ support for mobile health applications and their perception of the applications’ reliability. This indicates that pharmacists who perceive mobile apps as reliable are more likely to support their use in practice.

**Conclusion:**

Limited app use among Palestinian community pharmacists impacts medication safety, patient trust, and care quality. Adopting mobile tools can improve efficiency, reduce errors, and align pharmacy practice with modern standards, highlighting the need for future research.

## Introduction

Mobile health technologies have become integral to modern healthcare, offering healthcare professionals tools for clinical decision-making, patient management, and real-time access to drug information.^1^^,^^2^ The well-known adoption of smartphones and the rapid increase of mobile medical applications (apps) have revolutionized how healthcare providers access and utilize critical information.^2^ These apps support a wide range of functions, from providing clinical guidelines to enhancing disease management, making them essential in improving healthcare outcomes.^3^^,^^4^

Globally, the adoption of mobile health apps has been driven by their ability to simplify workflows, improve access to up-to-date clinical resources, and enhance communication between healthcare providers and patients. However, the successful integration of these technologies into healthcare systems varies widely, often influenced by resource availability, infrastructure, user training, and culture.^5^, ^6^, ^7^, ^8^

In Palestine, community pharmacists play a pivotal role in the healthcare system, often serving as the first point of contact for patients.^9^ Despite their essential contributions – such as medication dispensing, patient counseling, and drug therapy management – pharmacy practice in Palestine faces significant limitations.^10^ Many pharmacists operate with minimal technological infrastructure, relying on manual processes for inventory management and record keeping. Additionally, the sector is characterized by fragmented practices, where pharmacists must deal with high patient demand, unclear prescriptions by illegible handwriting, dosage double-checks, and price negotiations, often under time pressure.^11^, ^12^, ^13^ These challenges hinder the consistent delivery of high-quality pharmaceutical care.

Mobile health apps offer a potential solution to these challenges by equipping pharmacists with tools for faster and more accurate drug information retrieval, improved decision-making, and better communication with patients.^14^^,^^15^ However, their adoption in Palestine remains limited.

While mobile apps have been widely researched and implemented globally, studies specific to a low-resource setting like Palestine remain scarce. Insights from countries with similar healthcare constraints suggest that these tools can significantly improve workflow efficiency, patient outcomes, and service quality. However, the unique socioeconomic and healthcare environment in Palestine necessitates local research to explore the feasibility, acceptance, and challenges of apps adoption among community pharmacists.

This study aims to explore the perceptions, awareness, and challenges faced by community pharmacists in Palestine regarding mobile health apps. By identifying the barriers to adoption and assessing the feasibility of integrating these tools into practice. The findings of this study will contribute to the growing body of knowledge on mobile health technologies in resource-limited settings, offering practical recommendations for the digital transformation of community pharmacy practices in Palestine. Hence, enhancing healthcare delivery and patient outcomes.

## Methods

### Study Design and Setting

A cross-sectional online survey was conducted over 2 months in 2023 to explore the use of mobile medical applications among community pharmacists in Palestine. The study employed a quantitative design to gather data on pharmacists’ app usage, patterns, and perceived barriers to integrating mobile applications. The survey targeted community pharmacists actively practicing in Palestine, as they represent a critical link between healthcare systems and patients.

### Sampling and Sample Size

The target population included all registered community pharmacists in Palestine, estimated as approximately 4000 community pharmacists out of 9657 registered pharmacists in Palestine according to the Palestinian Pharmacy Association and Palestinian Ministry of Health, and around 1078 registered pharmacies in the West Bank, Palestine.^16^ Using a 95% confidence level, a 5% margin of error, and a 50% response rate, the minimum required sample size was calculated as 384 participants using Raosoft®.^17^ A total of 400 pharmacists completed the survey, achieving the target sample size. Since the survey was distributed openly via social media platforms, a response rate could not be calculated. However, the achieved sample size ensures adequate representation of the registered pharmacists in Palestine.

### Eligibility Criteria

Inclusion criteria: Licensed community pharmacists actively working in community pharmacies in Palestine and able to access the survey electronically.

Exclusion criteria: Pharmacists working outside community pharmacies, such as hospitals, companies, or academic institutions, as well as those not currently practicing.

To ensure that only the target population filled in the questionnaire, the survey link was distributed exclusively through verified pharmacist networks and social media groups restricted to licensed pharmacists in Palestine. Additionally, the questionnaire included a screening question confirming participants' licensure and active practice in community pharmacies. Respondents who did not meet these criteria were excluded from the study, ensuring that only eligible pharmacists participated.

### Data Collection

Data was collected using a structured, self-administered electronic questionnaire designed to be completed online as a Google Form®. The survey link was distributed through pharmacist networks and social media platforms. To ensure wide participation, reminders were periodically shared over the 2-month data collection period. Participation was entirely voluntary, and responses were collected anonymously to protect participant confidentiality.

### Questionnaire Development and Validation

The questionnaire was developed based on a comprehensive review of relevant literature and insights from previous studies on mobile health applications in pharmacy practice.

The questionnaire was designed to comprehensively explore various aspects of pharmacists’ demographics, practices, and perceptions regarding mobile health applications. It began with a section on demographic characteristics, gathering essential information such as gender, age, academic qualifications, and years of professional experience. This foundational data provided context for analyzing subgroup differences and identifying trends within the sample.

Subsequent sections focused on pharmacists' practice and information sources, examining the types of drug-related inquiries they frequently encountered and the resources they relied on to address these queries. This section sought to understand the interplay between traditional and digital resources in routine pharmacy practice.

To capture pharmacists’ perspectives on mobile health applications, the questionnaire included a section exploring their perceived advantages and reliability. This was followed by a segment on the usage of mobile devices and medical applications, collecting data on the ownership of smart devices, the types of medical apps installed, and the frequency of their use in daily practice.

The utilization of medical applications was further explored by investigating how often pharmacists used these tools to resolve patient inquiries and their perceived helpfulness in enhancing patient care. The questionnaire also delved into patient interactions and perceptions, focusing on how patients reacted to pharmacists’ use of mobile devices during consultations and the potential impact on trust.

Finally, the questionnaire addressed pharmacists’ attitudes toward mobile health applications. This section highlighted trust concerns and explored their willingness to recommend health apps to patients, providing valuable insights into the barriers that may hinder the broader adoption of mobile technologies in pharmacy practice.

The initial version of the questionnaire underwent face and content validation by a panel of 5 experts in pharmacy practice. The panel reviewed the questionnaire for clarity, relevance, and comprehensiveness, providing feedback that informed refinements to the final version. The finalized questionnaire consisted of 26 questions, employing multiple-choice and Likert scale formats, with some allowing multiple responses to capture a broader range of perspectives.

### Data Analysis

The data were analyzed using Statistical Package for Social Sciences (IBM SPSS Statistics ®21). Descriptive statistics, including frequencies and percentages, were calculated for categorical variables. For multiple-response questions, percentages were based on the number of participants selecting each option. Nonparametric techniques were utilized in the data analysis as these do not require a normal distribution in the identification of underlying trends. Associations between demographic questions and included Spearman's correlation, the p values were then measured; where a p-value of less than or equal to 0.05 was considered to be of statistical significance.

### Ethical Considerations

The study adhered to ethical standards for research involving human participants. Participation in the survey was voluntary, and all participants provided informed consent before completing the questionnaire. The survey was anonymous, and no identifying information was collected, ensuring participants’ privacy. The data collected were used solely for research purposes.

## Results

### Demographics and Professional Practice

A total of 400 licensed community pharmacists participated in the study, all actively practicing in community pharmacies across Palestine. The demographic and professional characteristics of the participants are summarized in Table 1, providing insights into their gender, age, marital status, educational qualifications, professional experience, work roles, and geographic distribution.

**Table 1:** Demographics and Professional Characteristics of Community Pharmacists.

	Frequency	Percent
**Gender**		
Male	137	34.3
Female	263	65.8
**Marital Status**		
Married	277	69.3
Single	111	27.8
Divorced/ Widowed	12	3
**Education**		
Bachelor	252	63
PharmD	78	19.5
Postgraduate	70	17.5
**Years of Experience**		
0–2 years	66	16.5
3–5 years	133	33.3
6–10 years	148	37
>10 years	53	13.3
**Occupation**		
Employee	382	95.5
Manager	18	4.5
**Work Shift**		
Morning	125	31.3
Afternoon	135	33.8
Evening	140	35
Frequency		
City	236	59
Village	125	31.3
Camp	39	9.8
**University**		
Palestinian University	273	68.3
Non-Palestinian University	127	31.8
**Area**		
Jenin	41	10.3
Tubas	33	8.3
Nablus	60	15
Salfit	24	6
Tulkarm	28	7
Ramallah	36	9
Jericho	30	7.5
Hebron	54	13.5
Bethlehem	31	7.8
Gaza	63	15.8

### Pharmacists’ Practice and Information Sources

Community Pharmacists frequently encounter a wide range of drug-related inquiries, pharmacists were asked about the types of inquiries they frequently encountered in daily practice that required consulting drug information resources, as well as how they ensured the accuracy of the information accessed. The results are summarized in Table 2.

**Table 2:** Inquiries Requiring Drug Information and Methods to Ensure Accuracy.

Question	Response Options	Frequency	Percentage
In your daily pharmacy practice, what types of inquiries frequently require you to consult a drug information resource? (multiple answers allowed)	Correct dosage and administration	392	98%
	Drug interactions	356	89%
	Side effects or adverse reactions	341	85.25%
	Indications	333	83.25%
	Contraindications	294	73.50%
	Special populations (e.g., pregnant, elderly, pediatric)	320	80%
	Alternative medications (due to unavailability)	280	70%

How do you ensure the accuracy of the information you consult? (multiple answers allowed)	I cross-check multiple resources	241	60.25%
	I rely on trusted sources	178	44.50%
	I consult with colleagues	154	38.50%
	I am unsure of its accuracy	27	6.75%

**Figure 1:**
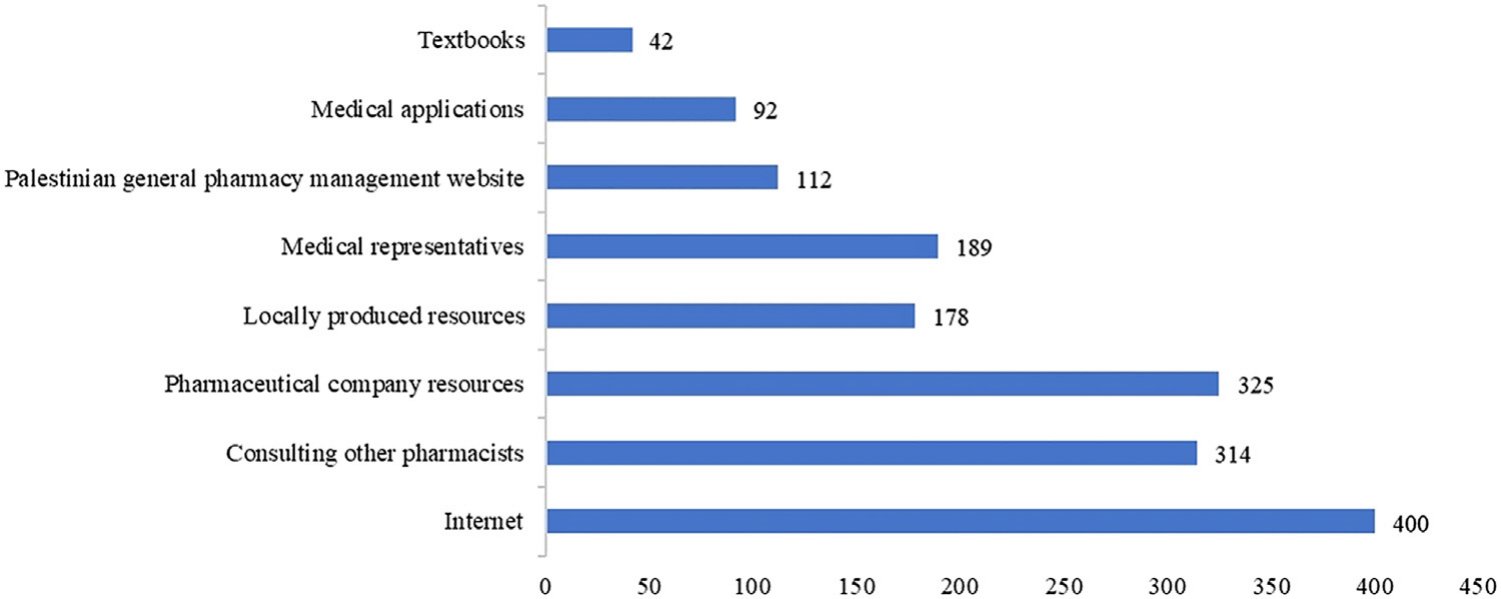
What is your most common source of information in the community pharmacy?

Pharmacists were asked to identify their most common sources of information in community pharmacy practice. The responses are presented in Figure 1.

### Use of Mobile Medical Applications

**Figure 2:**
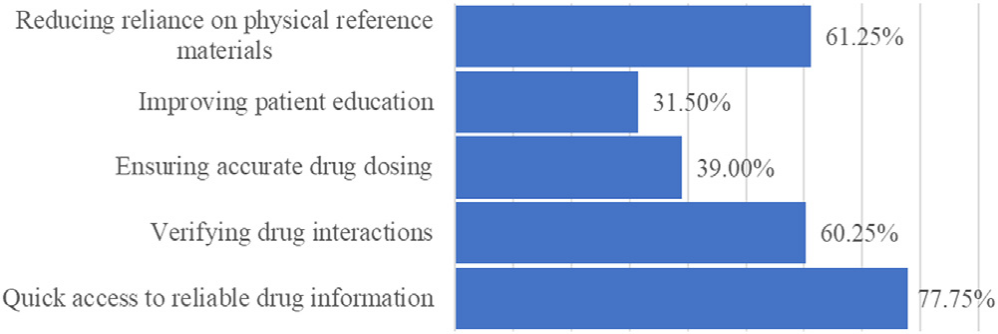
“In your opinion, what are the primary advantages of using medical applications in pharmacy practice?”

**Figure 3:**
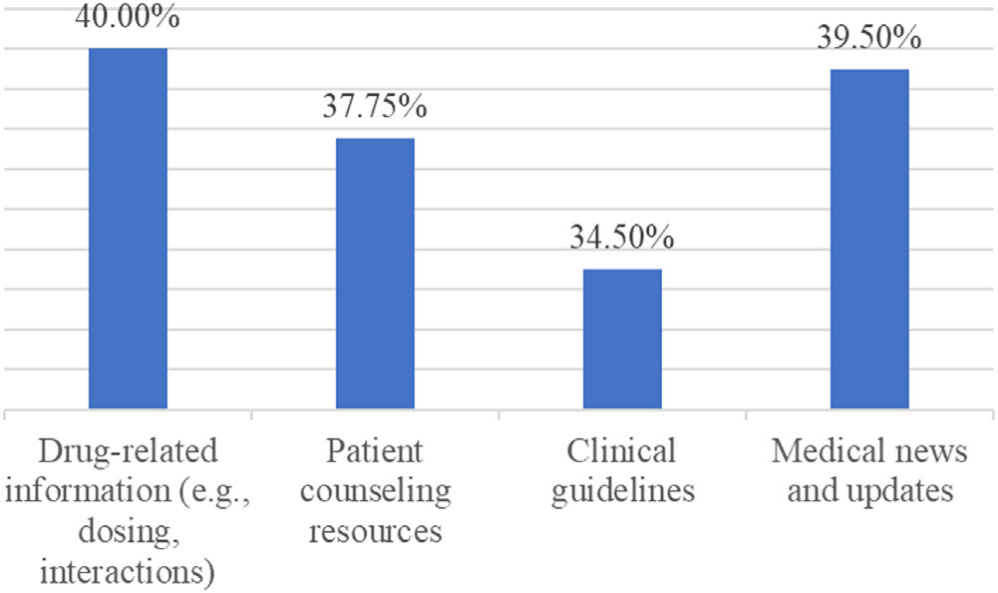
“In your opinion, Medical applications can be used to help the pharmacist by ...”

Pharmacists were asked to identify the primary advantages of using medical applications in pharmacy practice, with the results outlined in Figure 2. Also, pharmacists were asked their opinions on how medical applications can assist them in their practice, with the results presented in Figure 3.

Table 3 presents the responses to key questions in the questionnaire, categorized into distinct themes for clarity and coherence. These themes include the efficiency of using mobile applications to search for medical-related information, the importance of incorporating mobile application training into pharmacy education, and the frequency of use in patient interactions. Additionally, the table explores patient reactions to the use of mobile devices, the impact of medical applications on personal interaction during counseling, pharmacists’ support for recommending health applications to the public, and concerns regarding the potential loss of patient trust when using mobile devices during consultations. This thematic organization highlights the diverse perspectives of pharmacists on the integration of mobile health applications into their daily practice.

**Table 3:** Responses to Questionnaire Items on Pharmacists’ Perceptions, Practices, and Attitudes Toward Mobile Health Applications (N = 400).

Item	Frequency	Percent
**In your opinion, how long does it take to search a medical-related information by using medical applications?**		
Less than 30 seconds	21	5.3
30 seconds to 1 minute	85	21.3
1–2 minutes	112	28.0
2–5 minutes	144	36.0
More than 5 minutes	38	9.5
**Do you believe that incorporating training on medical applications into pharmacy education is important?**		
Very Important	139	34.8
Important	190	47.5
Somewhat Important	55	13.8
Not Important	16	4
**How often do you use the medical applications in front of patients?**		
Usually	84	21
Sometimes	82	20.5
Rarely	24	6
Never	210	52.5
**If you use a mobile device in front of a patient to check information, how do they usually respond?**		
Comfortable and Appreciative	21	5.3
Neutral, showing no particular reaction	44	11
Confused or Hesitant	56	14
Frustrated and not tolerant	47	11.8
I do not use a mobile device in front of patients	232	58
**Do your patients tend to repeat their questions to clarify doubts when you use a smart device in front of them?**		
Yes, frequently	26	6.5
Yes, sometimes	50	12.5
No, rarely or never	43	10.8
Not applicable - I do not use mobile in front of patients	281	70.3
**Do mobile health applications reduce personal interaction and the patient-centered approach during counseling?**		
Not at all – No impact or enhances interaction	166	41.5
Slightly – Minimal reduction in interaction	121	30.3
Moderately – Noticeable reduction in interaction	72	18
Significantly – Major reduction in interaction	34	8.5
Entirely – Completely replaces personal interaction	7	1.8
**Do you support the use of health applications on smartphones as a recommendation to the public?**		
Strongly support	86	21.5
Support	10	2.5
Neutral	149	37.3
Do not support	152	38
Strongly do not support	3	0.8
**The patients might lose trust in the pharmacist if the pharmacist checks their mobile device during a consultation.**		
Strongly agree	83	20.8
Agree	3	0.8
Neutral	10	2.5
Do not agree	149	37.3
Strongly do not agree	155	38.8

**Figure 4:**
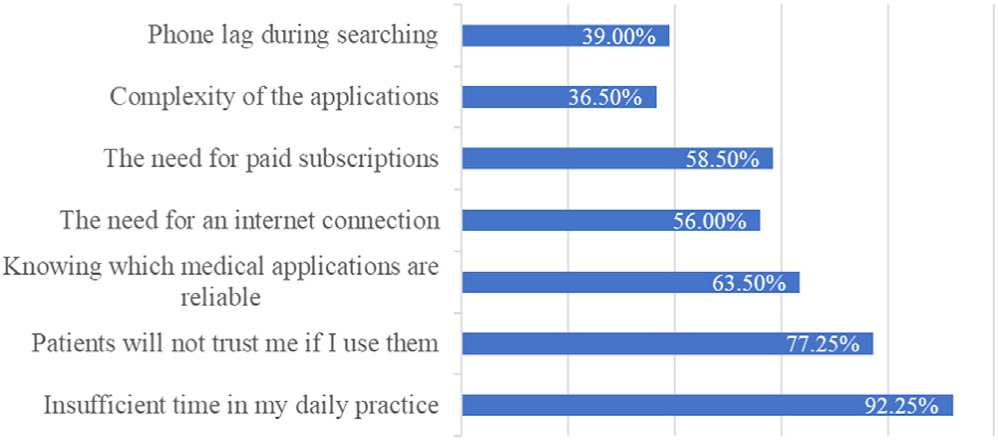
“What factors or challenges limit your use of medical applications in practice?”

The questionnaire explored factors limiting community pharmacists' use of medical applications. Figure 4 summarizes the main barriers.

### Relationships Between Pharmacists' Demographics and Perceptions of Mobile Applications

#### Time Required for Searching Information

Crosstabulations were performed to examine the relationship between demographic factors and the time required to search for medical information using mobile applications. The time varied slightly across experience levels, with pharmacists with more than 10 years of experience reporting the highest proportion of searches taking more than 5 minutes (17%). However, this difference was not statistically significant (*P* = 0.298). Similarly, pharmacists working in camps reported the longest search times (17.9%) compared to cities (20.3%) and villages (14.3%), though these differences were also not statistically significant (*P = 0*.629).

#### Perceptions of Training Importance

Perceptions of the importance of training in mobile applications were consistent across experience levels, with no significant variation observed (*P = 0*.790). Gender differences in the perceived importance of training were weakly significant (r_s_= 0.119, *P = 0*.017), with female pharmacists slightly more likely to value such training.

##### Gender and Patient Trust

No significant association was found between gender and perceived trust issues when using mobile devices (χ^2^=0.57, *P = 0*.45), indicating that both male and female pharmacists face similar challenges. However, a weakly significant linear-by-linear association (*P = 0*.017) suggested that female community pharmacists were slightly more concerned about potential trust loss when using mobile applications.

#### Experience and App Usage

Years of experience did not significantly impact perceptions of patient trust (χ^2^=5.80, *P = 0*.93) or the adoption of mobile apps. Early-career pharmacists (0–2 years) were less likely to use mobile applications in front of patients (57% "rarely" or "never") compared to those with 6–10 years of experience (57% "sometimes" or "usually").

#### Geographic Location and Patient Responses

Pharmacy location (city, village, or camp) did not significantly influence patient reactions to mobile device use (χ^2^=8.97, *P = 0*.34), suggesting consistent responses across geographic settings.

### Correlations Between Demographics and Perceptions

#### Education and Experience

Spearman's rank correlation analysis revealed a positive association between years of experience and educational background (r_s_ = 0.122, *P = 0*.015), suggesting a potential link between advanced education and longer professional experience.

#### App Support and Perceptions of Reliability

Perceived support for health applications was strongly correlated with the reliability of mobile applications as drug information resources (r_s_ = 0.522, *P <0*.001). There was also a significant negative correlation between app support and perceptions of longer search times (r_s_ = −0.219, *P <0*.001), indicating that pharmacists who perceived mobile applications as slower were less likely to support their use.

#### Patient Interaction and Trust

Favorable patient responses to mobile device use were positively correlated with trust in pharmacists (r_s_ = 0.121, *P = 0*.015). A negative correlation (r_s_ = −0.43, *P <0*.01) indicated that as the frequency of app use increased, perceived barriers decreased.

### Reliability Analysis

Multi-item constructs for "Barriers to App Use" demonstrated moderate reliability (Cronbach's Alpha = 0.68). A separate analysis of items related to app perceptions showed good reliability (Cronbach's Alpha = 0.831), with corrected item-total correlations ranging from 0.452 to 0.883. This indicates that the items were internally consistent in measuring pharmacists' perceptions and barriers.

## Discussion

Studies emphasized the role of smartphones in healthcare and their integration enhances clinical outcomes by providing immediate access to information.^18^ The integration of mobile health applications into Palestinian community pharmacy practice offers a unique perspective on how digital tools are reshaping healthcare delivery, exploring professional practice, and how cultural considerations can be explored.

Mobile apps hold significant promise as reference sources, particularly for quick access to drug information, verifying drug interactions, and ensuring accurate dosing. However, the results reveal that pharmacists in Palestine underutilize these tools, with only 66% recognizing their potential for quick access to reliable information and even fewer using them consistently in practice. The limited confidence in apps’ reliability is concerning, especially given the growing body of evidence, such as Mosa et al.^18^ which confirms the validity and usefulness of well-designed medical apps in healthcare.

Compared to traditional resources like pharmaceutical company materials or consultations with colleagues, mobile apps offer a more dynamic and accessible platform. Yet, their potential is hindered by skepticism about the information they provide. For instance, only 66% of pharmacists cross-check app information with other sources, a practice essential for ensuring reliability. This suggests that mobile apps are perceived as supplementary rather than primary tools, a view that contrasts with pharmacists in more developed settings, where apps like Medscape and UpToDate are widely trusted and integrated into daily workflows.^19^

### Community Pharmacists' Use of Mobile Medical Applications

The mere presence of digital tools does not mean their adoption, often due to lack of training, cultural perceptions, or infrastructural limitations.^20^ Aungst's review highlights the benefits of mobile medical applications, including portability, convenience, and instant access to information.^14^

While most pharmacists own smart devices and have medical apps installed, only a small percentage use these apps very frequently for patient inquiries, and even fewer find them helpful. This hesitancy could be due to a lack of familiarity, uncertainty about the reliability of apps, or discomfort in using them during consultations. The fact that many patients feel "confused" or "skeptical" when pharmacists consult apps will make the pharmacists not incorporate these tools into their practice.

Community Pharmacists in Palestine prioritize maintaining their perceived authority in front of patients, viewing visible reliance on apps as a potential threat to their professional image. This cultural effect was observed in other studies from other Middle Eastern countries, showing the importance of understanding how social expectations shape professional behavior.

This issue contrasts with findings in more developed contexts like the UK,^19^ where better-designed apps, combined with higher patient awareness, have led to greater acceptance and adoption of mobile tools in pharmacy practice. Higher adoption rates reported elsewhere in different contexts. For example, Ming et al. found that hospital pharmacists in Malaysia frequently used apps like Medscape, Micromedex, and Lexicomp to access drug information and improve efficiency in patient care.^21^ Similarly, Davies et al. noted that pharmacy apps are becoming a critical part of modern pharmacy practice, providing quick access to drug information and improving overall service delivery.^22^

### Patient Perception and Trust

The reported skepticism and discomfort among patients when community pharmacists use mobile apps during consultations are particularly striking. These findings suggest a lack of trust in technology as a reliable adjunct to professional knowledge. Interestingly, this reaction contrasts with attitudes in more technologically integrated healthcare systems, where patients often expect healthcare professionals to use digital tools.^23^

This discrepancy raises questions about how patients in Palestine perceive the role of technology in healthcare. Are they skeptical because they view pharmacists as “all-knowing,” or does this skepticism stem from a broader distrust in digital solutions? Further research into patient attitudes could illuminate whether public education campaigns could shift these perceptions, emphasizing that digital tools support rather than replace professional judgment.

The vast majority of pharmacists believed that using mobile devices in front of patients could harm their professional image, with patients possibly perceiving them as less knowledgeable. This concern is consistent with findings from Jordan, where Al Subeh et al. reported that both students and preceptors shared concerns over how the use of mobile apps might affect patient perceptions.^24^ Although trust takes time to build, maintaining it needs effort. The dynamic relationship between pharmacists and patients has been studied thoroughly 25, 26, 27, 28, 29. The context of pharmacy practice in Palestine remains unique, and this is why community pharmacists mostly agree on the trust of patients. In a qualitative study in Palestine seeking the barriers to patient counseling, patients noted these reasons for not wanting to approach the pharmacist “not viewed as a reliable resource, lack of confidence in their abilities, greater trust in the physician to provide information, written information is sufficient.”^30^ This highly aligns with the findings of this study and another study by Sawalha et al.^31^ investigated the barriers to patient counseling in Palestine by pharmacists. The reasons were “The participants did not have enough time to listen, the participants did not want to listen for whatever reason(s) they had, The participants have doubts regarding the pharmacists’ knowledge, and were not sure they could be trusted. The participants thought it's better to obtain the information they need about drugs or diseases from the treating physicians.”

The concern that patients might lose trust in pharmacists who use mobile devices during consultations is not unique to Palestine. Payne et al. found similar concerns among medical students and junior doctors in the UK, who feared that using smartphones in front of patients could be perceived as unprofessional. In Palestine, where personal interaction plays a significant role in healthcare, pharmacists' hesitation to use mobile apps in front of patients reflects the cultural emphasis on maintaining direct, personal communication with patients.

However, as mobile technology becomes more integrated into healthcare, educating both pharmacists and patients about the value of these tools will be essential. Mobile apps can improve accuracy and efficiency, and by increasing patient awareness of these benefits, pharmacists may be able to build trust while using these tools during consultations.

### Challenges in Pharmacy Practice in Palestine

Palestinian community pharmacists face challenges such as time constraints, the need for internet connectivity, and the difficulty in identifying reliable applications. In another context, in Jordan, Al Subeh et al. investigated the use of medical apps among pharmacy students and preceptors.^19^^,^^24^ Pharmacy students, preceptors, and faculty members generally recognize the value of medical applications in pharmacy education and practice. This group of pharmacists do not interact directly with the patients and students do not feel the real situation and they are acting upon their expectations. Also, preceptors are working at hospitals and optimal conditions, not community pharmacies, so they have much more time to consult medical applications.

Kayyali et al. found that similar barriers like lack of time and awareness, hindered pharmacists in the UK from regularly adopting mobile apps.^19^ Likewise, in Malaysia, pharmacists faced issues related to app subscription costs and unreliable internet access, challenges that resonate with those faced by Palestinian pharmacists.^21^ These findings align with the broader context of pharmacy practice in Palestine, which remains under-resourced compared to other Middle Eastern and Western countries.^32^

A study from Oman found that their pharmacists still rely heavily on standard books for drug information,^33^ contrasting to not relying on textbooks among community pharmacists in Palestine. This shift towards digital resources in Palestine likely reflects the increasing availability of online information and the need for real-time updates in a rapidly evolving healthcare landscape.

Pharmacy practice in Palestine faces numerous challenges. According to Sweileh et al., while the number of pharmacists has grown significantly, the sector is still underdeveloped, with limited access to clinical pharmacy services, automation, or advanced technologies.^10^ Similarly, Jaradat and Sweileh highlighted that many community pharmacists rely on manufacturer brochures and medical representatives as primary sources of drug information, rather than digital tools.^10^^,^^32^ This reliance on non-digital sources further complicates the integration of mobile technologies into daily practice.

Compounding services, another critical area of community pharmacy practice in Palestine, face similar challenges. Zaid et al. found that while 72.2% of pharmacists offer compounding services, these represent only a small percentage of total prescriptions due to concerns over the quality of compounded medicines.^34^ This lack of trust in pharmaceutical compounding can be associated with the hesitation to adopt mobile medical apps, highlighting a broader pattern of reluctance to fully embrace new technologies in Palestinian pharmacies.

The attitudes of Palestinian community pharmacists toward generic medicines also provide insights into their approach to technology. Shraim et al. found that while the majority of pharmacists supported generic substitution, their knowledge of technical aspects like bioequivalence and pharmacokinetics was limited.^12^ This lack of technical understanding could contribute to the hesitation in adopting digital tools, which often provide detailed and complex data on drug interactions, side effects, and generics.

### Opportunities for Adoption and Improvement

The majority of community pharmacists in Palestine believe that medical applications could improve their performance. This finding is consistent with research from Jordan and Malaysia, where pharmacists have reported that mobile apps enhance efficiency and reduce the time needed to answer patient inquiries.^21^^,^^24^ However, the successful adoption of these tools will require addressing the structural barriers identified in this study.

One promising solution is the wider adoption of free, accessible apps such as Medscape. Ming et al. emphasized Medscape's popularity in Malaysia due to its ease of use and accessibility, making it an ideal tool for pharmacists working in low-resource environments.^21^ By increasing awareness of such apps and ensuring reliable internet access, the barriers related to cost and availability can be mitigated.

The rise of pharmacy practice research in the Middle East, as noted by Obaid et al., offers further opportunities for Palestine to develop tailored strategies for adopting mobile health technologies.^35^ Developing a specific research agenda focused on improving digital literacy and the integration of mobile apps into pharmacy practice could position Palestine as a leader in the region and help pharmacists better serve their communities.

### Implications for Pharmacy Practice in Palestine

The underutilization of mobile apps by community pharmacists in Palestine has direct implications for medication error rates, patient safety, and the overall quality of pharmaceutical care. The lack of confidence in using mobile apps as reliable resources could lead to missed opportunities for preventing medication errors.^36^ Apps designed for verifying drug interactions, proper dosages, and contraindications are proven tools for reducing errors, as shown in studies like Ventola (2014).^20^ Without effective integration of these tools, pharmacists may rely on incomplete or outdated information, increasing the risk of prescribing or dispensing errors. This is particularly concerning in community pharmacy settings where pharmacists often operate independently without immediate access to institutional guidelines or multidisciplinary teams.

The limited use of apps in professional development and patient consultations suggests that community pharmacists are not fully engaging with the latest clinical resources. This can impact the quality of pharmaceutical care, as pharmacists may struggle to provide evidence-based answers to patient inquiries. For example, patients with complex needs, such as pregnant women or elderly individuals, may require nuanced drug recommendations that apps could support. The absence of these tools in practice might result in generic advice that does not fully address individual patient needs.

Patient skepticism toward the use of apps during consultations (as reported in the study) highlights a gap in communication and trust. When pharmacists hesitate to explain the role of mobile tools in ensuring accurate and timely information, it can create doubts about their competence. Studies like Gregory and Austin (2021) emphasize that transparent communication and patient education are critical to fostering trust.^25^ Without addressing this dynamic, community pharmacists may find it harder to build the patient relationships necessary for effective care delivery.

Training community pharmacists on the effective use of mobile apps is essential to improving their confidence and integrating these tools into daily practice. Such training programs should focus on familiarizing community pharmacists with app functionalities, ensuring they can quickly access reliable information on drug interactions, dosages, and contraindications. Additionally, training should include strategies for incorporating apps into patient consultations in a way that builds trust, such as explaining their purpose and benefits to patients. By equipping community pharmacists with these skills, training programs can enhance the quality of care, reduce medication errors, and make community pharmacists more efficient in their roles.^36^

As noted by Almarri and Bhatti, consumers in the United Arab Emirates were largely unaware of mobile health apps, but once exposed to them, they found them helpful for managing their health.^37^ Similar educational campaigns in Palestine could increase patient trust in mobile technologies, allowing pharmacists to use these tools more confidently during consultations.

### Limitations

The reliance on self-reported data may have introduced some bias, as pharmacists could either overstate or understate their use of these technologies. Moreover, the study was conducted among community pharmacists only, excluding those in hospital or academic settings, which may limit the applicability of the findings to the wider pharmacist population in Palestine. The sample included pharmacists from all governorates in Palestine, ensuring geographic representation. However, the use of online distribution could have led to over- or under-representation of specific regions. This made the possibility of non-community pharmacists had access to the study tool and may inadvertently filled it, regardless of the survey question that restricts them, this possibility had to be acknowledged in the limitations. Further research with targeted sampling at physical community pharmacy locations is recommended for more robust geographic representativeness.

## Conclusion

While mobile medical applications hold the potential to enhance pharmacy practice, their integration remains limited among Palestinian community pharmacists. The limited confidence and utilization of mobile apps among pharmacists in Palestine have significant implications for medication safety, patient trust, and the quality of pharmaceutical care. Addressing these issues requires targeted training to improve pharmacists' familiarity with app functionalities, as well as public education to reduce skepticism and build trust in their use during consultations. By adopting mobile tools to provide accurate and timely drug information, pharmacists can enhance their efficiency, reduce medication errors, and deliver higher-quality care. Integrating these solutions into pharmacy practice can bridge existing gaps and align the profession with modern healthcare standards. This study provides a foundation for future research into optimizing the use of mobile health tools to strengthen pharmacy practices and patient care, ultimately reducing medication errors.

## Declaration of Generative AI and AI-assisted Technologies in the Writing Process

During the preparation of this work, the author(s) used ChatGPT in order to improve the readability and understanding of the content. After using this tool/service, the author(s) reviewed and edited the content as needed and take(s) full responsibility for the content of the publication.

## Declaration of competing interest

The authors declare that they have no known competing financial interests or personal relationships that could have appeared to influence the work reported in this paper.
